# The Evolution of Psychological Distress Levels in University Students in Spain during Different Stages of the COVID-19 Pandemic: Risk and Protective Factors

**DOI:** 10.3390/ejihpe13110180

**Published:** 2023-11-09

**Authors:** María Pilar Matud, Jesús Zueco, Maria José Del Pino-Espejo, Demelsa Fortes, María Ángeles Beleña, Cristina Santos, Amelia Díaz

**Affiliations:** 1 Department of Clinical Psychology, Psychobiology and Methodology, University of La Laguna, 38200 La Laguna, Spain; pmatud@edu.ull.es (M.P.M.); demel81@hotmail.com (D.F.); alu0100892697@ull.edu.es (C.S.); 2 Department of Microbiology, University of Valencia, 46100 Valencia, Spain; jesus.zueco@uv.es; 3 Department of Sociology, University of Pablo de Olavide, 41013 Sevilla, Spain; mjpinesp@upo.es; 4 Department of Personality, Assessment and Psychological Treatments, University of Valencia, 46010 Valencia, Spain; mangeles.belena@uv.es

**Keywords:** psychological distress, COVID-19-associated stressful events, self-esteem, social support, university students

## Abstract

The present study assesses the evolution of stressful events and psychological distress in male and female students over three different time periods of the COVID-19 pandemic in Spain: the initial “lockdown”, with no face-to-face teaching; the “new normality” period, when classes were resumed; and two years after the first wave of the pandemic. The participants were 1200 Spanish university students who were assessed for psychological distress, COVID-19-associated stressful events, social support, and self-esteem. Female students reported more stressful events and higher levels of psychological distress than male students during the “lockdown” and “new normality” time periods of the first wave of the pandemic. However, these differences disappeared in the third period tested, two years after the first wave of the pandemic, with female and male students showing no differences in psychological distress or in the number of stressful events. The main risk predictors of psychological distress during the first wave of the pandemic were lower self-esteem and having suffered a high number of stressful events. The last variable, number of stressful events associated with COVID-19, lost most its effect two years later, when only self-esteem presented a strong and highly significant predictive role.

## 1. Introduction

The World Health Organization (WHO) declared the COVID-19 disease a pandemic on 11 March 2020 [1], and the disease quickly became a global health crisis [2], generating stress throughout the population [3]. A pandemic situation induces high stress levels which are the logical consequence of the perceived risk of infection and death [4]. The COVID-19 outbreak can be considered an acute stressful situation which implies a potential danger to oneself and to close relatives [5]. Furthermore, in the case of COVID-19, the immediate measures introduced to curb the outbreak involved changes in lifestyle that could be a source of stress, in addition to the economic, employment, and educational consequences of the pandemic [6]. All this, together with the unpredictability of COVID-19, could induce stress in the population [7]. Although studies have shown very different rates in the prevalence of stress, varying according to the kind of stress evaluated and the sociodemographic characteristics of the sample, most of them found high stress levels in women and young people [8,9] as well as in healthcare workers [10]. De Sousa et al. [10] published a meta-review of prevalence meta-analyses that found that the global prevalence of stress was 36.12%, whilst that of psychophysiological stress was 31.99% and 20.67% in the general population and 37.74% in healthcare workers. But diversity in stress rates has also been found within this population. Thus, in a meta-analysis that included 40 studies published between January and September 2020 wherein stress in nurses was studied, it was found that the overall pooled period estimate varied between 10% and 84%, with the overall prevalence of stress being 43% [11]. Several studies conducted in different countries during the pandemic showed that students were among the groups with the highest level of stress [8], with a study carried out in Saudi Arabia in a random sample of 2034 university students showing that 76.84% suffered stress [12]. Studies carried out during the COVID-19 pandemic in Spain showed that the more frequent symptoms were associated with depression and anxiety, with women and young people presenting the worst mental health outcome [13,14].

Although stress is expected to increase during a pandemic, there is justified concern about the long-term effects of high stress levels [7]. In developed nations, stress is a major contributor to population suffering and is linked, directly and indirectly, to a range of human diseases that contribute to morbidity and mortality [15].

### 1.1. The Impact of the First Wave of COVID-19 on University Students

The first wave of COVID-19 severely affected Spain, with a very high number of cases and high mortality [16]. The state of alarm, commonly referred as the “lockdown”, was introduced on 15 March 2020. Spain had a strict lockdown with the suspension of any non-essential activity. Outdoor activities were forbidden, telework was imposed, and not even outdoor walks were permitted. In the educational system, the preventive measures included the suspension of face-to-face teaching in all educational centres and levels. As consequence of these measures, Spain suffered the most drastic reduction in human mobility among eight European countries, as determined using mobile positioning data [17]. Although these measures began to ease in April, the lockdown was extended until 21 June 2020, when the “new normality” period began, which was in effect up to 25 October 2020. At this time, most activities were permitted under the condition that social distancing of 1.5 m could be kept between individuals to prevent the spread of the virus.

The closure of educational centres was particularly stressful for students, who experienced a sudden change in lifestyle. In addition to changing their way of learning and assessment, which was temporarily moved online, they also lost their university environment, which impacted their academic and personal well-being [18], with evidence showing that the mental health of students was particularly affected [19].

It has been suggested that the changes induced by COVID-19 were even more pronounced for students at universities [18,20]. The closure of campuses forced most students to move back into their parents’ home, with the consequent cancellation of activities such as lectures, discussions, presentations, group work, sport activities, and social gatherings, and in some cases the addition of other household responsibilities, such as home-schooling their siblings, caring for relatives, or working to help economically support their family [21].

It has also been suggested that university students may have experienced specific stressors [22], including the above-mentioned changes in place of residence, loss of income, and changes to their study schedule. In a study carried out during the spring semester of 2020 focused on the academic and psychosocial frustrations faced by university students, it was found that COVID-19 had negatively influenced their academic well-being, highlighting frustrations with technology, classroom work, research, family relations, and struggles in social, emotional, behavioural, and financial areas [18]. Son et al. [23] found very high percentages of stressors associated with COVID-19 in university students, highlighting fear and worry for their health and their loved ones (91%), difficulty concentrating (89%), sleeping problems (86%), and decreased social interaction (82%).

All these stressors represented an additional burden for the students’ mental health during their development as emerging adults, which was already known to pose a higher risk of mental health problems before the pandemic. Emerging adulthood is a stressful, critical stage in which important changes in the life of individuals occur [24], including leaving home, finishing education, finding employment, establishing couple relationships, and becoming more independent and responsible in many important areas [25,26]. Accordingly, any additional distress suffered at this stage in life represents an important risk for the well-being of young adults, and this is even more pronounced in the case of women, who in many studies report higher distress than men [27,28], psychological distress being defined as a combination of symptoms including depression, anxiety, and behavioural problems [29].

Many of these problems begin in childhood and adolescence and, in some countries, it had already been observed that mental health issues in adolescents had increased even before COVID-19 [30]. University years have also been found to frequently be the period during which many mental disorders manifest for the first time [31], and psychological distress, as an important mental health problem among university students, has been associated with suicidal behaviour [32]. Consequently, it can be assumed that experiencing stressors during COVID-19 may have exacerbated existing mental difficulties in students within this period [22]. Studies on the psychological impact of the pandemic carried out in different countries have confirmed that the impact has been particularly high on students [33], with most of the above studies and many others also showing that the impact has been greater in female students than in male students [16,34].

### 1.2. COVID-19 and Psychological Distress: Protective and Risk Factors

Since the introduction of the Lazarus and Folkman Stress and Coping Theory [35], the role of stress in many human situations has been widely studied [36]. In this context, COVID-19 has undeniably acted as an acute and strong source of stress. There is abundant proof that the pandemic was a potentially stressful situation that generated distress in many people, as described in previous paragraphs [2,37,38]. However, the Lazarus and Folkman theory proposes not only risk factors but also protective factors which decrease the negative effects of stress. Among these, social support occupies a prominent position [39]. Social support is a protective factor that provides emotional support such as empathy, care, trust or listening, whilst instrumental support provides tangible elements such as money, time, or help; finally, informative support provides advice, information, or guidance [40]. Social support is among the factors that have been shown to protect against psychological distress during the pandemic, with students who have greater social support exhibiting lower levels of distress [2,34,41]. Gender has also been found to regulate the role of social support, as it is more important for women than for men in the late stages of development, adolescence and early adulthood [42]. Additionally, self-esteem is another factor that has been shown to be a protective factor against psychological distress during the pandemic, having been found to act as a buffer against threats to mental health [43]. Self-esteem was defined by Rosenberg as the positive or negative attitudes toward the self [44]. Social support and self-esteem have been broadly discussed in psychological research at any stage of development [42], with some studies having shown that social support is a good predictor of self-esteem in university students [45,46].

### 1.3. The Present Study

In the present study, we studied the relevance of direct experience in stressful events associated with COVID-19 as a risk factor, against the protective role of social support and self-esteem in psychological distress, in male and female university students during three time periods of the COVID-19 pandemic in Spain: the highly restrictive lockdown period, in which there were no face-to-face classes; the new normality period in October 2020, when face-to-face classes were restarted; and two years later (from October 2022 to February 2023). The reason for selecting two time periods in the first wave of the pandemic was to try to ascertain the impact of the strict lockdown and the more permissive new normality on the mental health of university students, whilst the third time period, two years later, was chosen to determine if the psychological distress levels had returned to normal by comparing our results with published studies performed just before the pandemic.

The following hypotheses were tested:

**Hypothesis** **1.** *Female students will report higher levels of psychological distress than male students in all time periods evaluated.*

**Hypothesis** **2.** *The psychological distress levels of both male and female students will be the highest during the period of the strict “lockdown”, lower in the “new normality” period, and the lowest two years later. Psychological distress levels during this third period will be similar to levels before the pandemic.*

**Hypothesis** **3.** *Students who have experienced more stressful events during the COVID-19 pandemic will show more psychological distress during the first wave of COVID-19, but this will decrease two years later.*

**Hypothesis** **4.** *Having experienced more experienced stressful events related to COVID-19 will predict higher psychological distress levels*.

**Hypothesis** **5.** *Higher social support will predict lower psychological distress levels.*

**Hypothesis** **6.** *Higher self-esteem will predict lower psychological distress levels.*

## 2. Materials and Methods

### 2.1. Participants, Design, and Procedure

The participants were 1200 students, of whom 47.3% were male and 52.7% were female. Participants’ age ranged between 18 and 27, with the mean for male students being 21.03 years (*SD* = 2.35) and that for female students being 21.21 (*SD* = 2.10), a difference which is not statistically significant: *t*(1,1198) = −1.36, *p* = 0.174. Most of the participants (74.8%) did not have a partner and 25.2% did. There were no statistically significant differences in the percentage of male and female students with and without a partner: χ^2^(1, *N* = 1200) = 0.002, *p* = 0.968. Data from 400 participants were obtained in June 2020, when the lockdown was still in effect in Spain; another 400 participants in October 2020, during the time of new normality in Spain, when face-to-face learning had resumed; and the last 400 participants two years later, from October 2022 to February 2023, a few months before the end of the COVID-19 pandemic was declared on the 5 of May 2023.

Samples were selected from a larger sample to assess the psychological impact of the COVID-19 pandemic during the three time periods cited. The following inclusion criteria, apart from the fact that the questionnaires had been completed in the time periods described, were used: (1) participating students had no children and (2) male and female students were equivalent in sociodemographic characteristics (age, living with/without a partner). The data for the first 400 university students obtained in the lockdown period determined the sociographic variables of the students in the new normality and the two-years-later time periods.

This study had a repeated cross-sectional design with different participants matched on key sample features, during three time periods. In order to prevent participants answering the questionnaires more than once during the time period, and as a way to ensure participants’ anonymity, a code with 8 digits was assigned to each participant.

Contact with the participants was made through the social networks of researchers and students from three Spanish universities: the University of La Laguna, University of Valencia, and Pablo de Olavide University of Seville. Participation was voluntarily and participants did not receive remuneration. Students who voluntarily collaborated in data collection were given 1.5 credits. The sample was obtained using a convenience and snowballing procedure. An online Google Form survey was used to obtain the data. Informed consent was obtained from all participants involved in this study, who could stop participating at any moment. The Ethical Guidelines of the American Psychological Association (APA) were adhered to throughout this study, and the procedures performed were in accordance with the ethical standards of the 1964 Helsinki Declaration and its further amendments or comparable ethical standards. The research was also approved by the Ethics Commission for Research with Human Beings (CEIH) of the University of La Laguna (code 21/8–6).

### 2.2. Measures

#### 2.2.1. Psychological Distress

The measurement of psychological distress was based on the use of the 12-item General Health Questionnaire (GHQ) [47]. Scoring was based on the Likert scale (0-1-2-3) and the Standard GHQ scoring system (0-0-1-1), with the highest score indicating greater distress. The Likert score was used in all the analyses, except for the calculation of the percentage of people with and without distress, where the Standard GHQ score was used. As reported by Lundin et al. [48], the best cut-off period to differentiate cases of distress from cases where distress is not present for the GHQ Index was ≥4. In this study, Cronbach’s alpha for the 12 items scored based on the Likert method was 0.88, and that for the Standard GHQ method was 0.87.

#### 2.2.2. Stressful Events during the COVID-19 Pandemic

To find out if students had experienced additional stressors to those triggered by the pandemic situation, they were asked if they had experienced the following events and/or losses during the pandemic: (1) economic problems, (2) serious arguments with their family, (3) serious arguments with their partner, (4) illness of relatives and/or loved ones, (5) death of relatives and/or loved ones, (6) and personal illness. In addition, a space was provided for people to freely write any other event or loss that had occurred during the pandemic.

#### 2.2.3. Perceived Social Support

Perceived social support was assessed using the Social Support Scale [49]. The scale is made up of 12 items using a 4-period Likert-type scale ranging from 0 (never) to 3 (always) for rating the responses. In the sample of this study, Cronbach’s alpha for perceived social support was 0.92.

#### 2.2.4. Self-Esteem

The Rosenberg Self-Esteem Scale [50] was used to evaluate self-esteem. Ten items made up the scale, and these are rated using a 4-period Likert scale ranging from 0 (strongly agree) to 3 (strongly disagree). In this study, Cronbach’s alpha was 0.86.

#### 2.2.5. Sociodemographic Variables

Participants were asked about their age, gender, and whether or not they lived with a partner.

### 2.3. Data Analysis

IBM SPSS, version 23.0, was used for data analysis. Differences between male and female students for each time period, and across the three time periods, were obtained via performing an analysis of variance between subjects (ANOVA) in which the independent variables were gender and time period, whilst the dependent variables were psychological distress in the first ANOVA and the number of stressful events experienced during the pandemic in the second one. The ANOVA allowed us to determine if gender interacted with the time period; thus, we were able to find out if the effect of gender differed throughout the pandemic, in addition to determining the main effects of gender and time periods. To determine the relevance of the number of stressful events, self-esteem, and social support in psychological distress in male and female students and in each time period, six hierarchical multiple regression analyses were carried out. Age was entered in step 1, social support in step 2, the number of stressful events in step 3, and self-esteem in step 4. Hierarchical multiple regression analyses were performed separately for male and female students and for each time period. Hierarchical regression analyses allowed us to determine step by step the relevance that age, number of stressful events, self-esteem, and social support had as protective and risk factors for psychological distress in the three study periods for women and men. Finally, the program G*Power 3.1.9.7 [51] was used to assess effect size and statistical power in statistically significant results.

## 3. Results

### 3.1. Differences in Psychological Distress and COVID-19-Associated Stressful Events

The ANOVA performed using gender and time period as factors and the psychological distress score as the dependent variable showed that the interaction of gender X time period was statistically significant: *F*(1,1194) = 4.57, *p* = 0.011, η^2^partial = 0.008, *f* = 0.09, 1 − β = 0.75; consequently, analyses were performed independently for each time period and gender. The ANOVA performed in June 2020 showed that the main effects of gender were statistically significant: *F*(1,398) = 21.27, *p* < 0.001, η^2^partial = 0.051, *f* = 0.23, 1 − β = 1. The mean score of female students in psychological distress (*M* = 18.16, *SD* = 6.80) was higher than that of male students (*M* = 15.13, *SD* = 6.30). The ANOVA performed in October 2020 showed that the main effects of gender were also statistically significant: F(1,398) = 20.52, *p* < 0.001, η^2^partial = 0.049, *f* = 0.23, 1 − β = 1. The mean score of female students in psychological distress (*M* = 18.42, *SD* = 6.96) was higher than that of males (*M* = 15.33, *SD* = 6.77). Finally, the ANOVA performed two years after the first wave of the pandemic did not show statistically significant differences for gender: F(1,398) = 0.82, *p* = 0.37. The mean score of female students in psychological distress was 14.75 (*SD* = 6.57), whilst that of male students was 14.15 (*SD* = 6.47). In the ANOVA in which the time period was introduced as a factor, no statistically significant differences were found in the male student sample: F(2,564) = 1.77, *p* = 0.17. However, in the female student sample, statistically significant differences were found when time period was introduced as a factor: F(2,630) = 19.40, *p* < 0.001, η^2^partial = 0.058, *f* = 0.25, 1 − β = 1. Post hoc analyses with Bonferroni adjustment showed that women had greater psychological distress (*p* < 0.001) in the two testing sessions carried out during the first wave of the pandemic than in the one carried out two years later.

Using a threshold of ≥4 in the GHQ score, the prevalence of psychological distress in female students was 74.2% and in male students 57.7% during the first wave of the pandemic, a difference that was statistically significant: χ^2^(1, *N* = 800) = 24.32, *p* < 0.001. In the lockdown period, the prevalence of psychological distress in female students was 73.9% and in male students 55.6%, a difference that was statistically significant: χ^2^(1, *N* = 400) = 14.85, *p* < 0.001. During the new normality period, the prevalence of psychological distress in female students was 74.4% and in male students 59.8%, a difference that was statistically significant: χ^2^(1, *N* = 400) = 9.71, *p* = 0.002. Two years after the first wave of the pandemic, the prevalence of psychological distress in female students was 51.2% and in male students 52.4%, a difference that was not statistically significant: χ^2^(1, *N* = 400) = 0.057, *p* = 0.811. There were no statistically significant differences in the rates of psychological distress between the three time periods (χ^2^(2, *N* = 567) = 2.11, *p* = 0.347) in the male student sample, whilst the differences in the rates of psychological distress between the three research times periods were statistically significant in the female student sample: χ^2^(2, *N* = 633) = 33.38, *p* ≤ 0.001.

**Figure 1:**
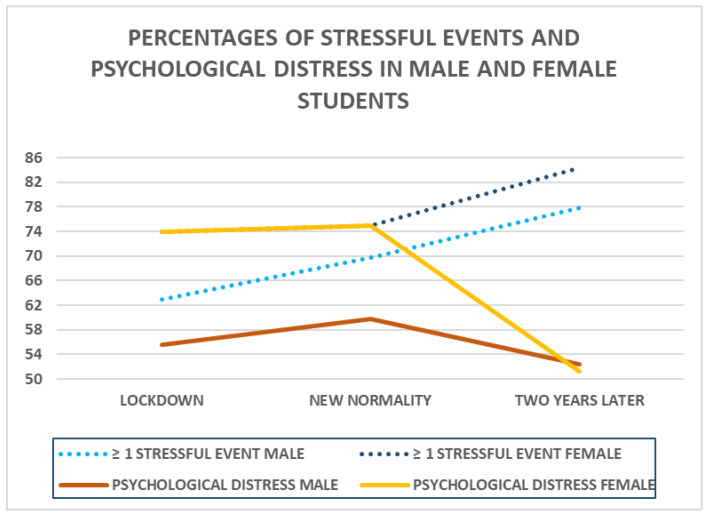
Percentage of male and female university students who experienced ≥ 1 stressful event and scores ≥ 4 in psychological distress.

The total number of stressful events during the first wave of the pandemic in Spain in the sample ranged from 0, for 29.6% of the sample, to 6, for two cases only. Accordingly, the mean, standard deviation, and median were, respectively, 1.39, 1.39, and 1. The ANOVA performed using gender and the time period as factors and the total number of stressful events during the COVID-19 pandemic as a dependent variable showed that the main effect of gender was statistically significant (F(1,796) = 15.31, *p* < 0.001, η^2^partial = 0.019, *f* = 0.14, 1 − β = 0.99), as was that of the time period: F(1,796) = 5.83, *p* = 0.016, η^2^partial = 0.007; *f* = 0.13; 1 − β = 0.95. However, the interaction of gender x time was not statistically significant: F(1,796) = 0.01, *p* = 0.97. The mean score for the number of stressful events in the female students was 1.55 (*SD* = 1.32), and that for the male students was 1.21 (*SD* = 1.19). The mean score for the number of stressful events in the lockdown period was 1.28 (*SD* = 1.20), in the new normality period 1.50 (*SD* = 1.33), and two years later 2.07 (*SD* = 1.56). Two years after the first wave of the pandemic, only 75 participants (18%) reported zero stressful events, and 4 (1%) reported the maximum score, six. At this time, the accumulative effect in the three time periods is evident. In the ANOVA performed two years after the first wave of the pandemic, no statistically significant differences were found between female and male students in number of stressful events: *F*(1,398) = 0.06, *p* = 0.810. The mean score for female students was 2.08 (*SD* = 1.52) and the mean score for male students was 2.05 (*SD* = 1.61). Figure 1 shows the percentages of male and female participants who experienced ≥ 1 stressful events and those reporting a psychological distress score ≥ 4 for the three time periods studied. The percentages of female students who experienced ≥1 stressful events and those reporting a psychological distress score ≥ 4 were the same in the lockdown and the new normality period, at 73.9%/73.9% and 74.9%/74.9%, respectively.

The most frequently cited event during the first wave of the pandemic was serious arguments with one’s family, which was cited by 42.4% of the female students (40% during the lockdown and 44% in the new normality period) and 32.0% of the male students (29% during the lockdown and 35% during the new normality period), a difference that was statistically significant: χ^2^(1, *N* = 800) = 9.21, *p* = 0.002. The second most frequent event was the illness of relatives and/or loved ones, which was cited by 32.9% of the female students and 24.9% of the male students, a difference that was statistically significant: χ^2^(1, *N* = 800) = 6.29, *p* = 0.012. Economic problems followed in frequency, being cited by 31.5% of the female students and 22.2% of the male students, a difference that was statistically significant: χ^2^(1, *N* = 800) = 8.71, *p* = 0.003. The next most cited problem was the death of one or more relatives or loved ones, which was cited by 21.6% of the female students and 16.9% of the male students, a difference that was not statistically significant: χ^2^(1, *N* = 800) = 2.74, *p* = 0.098. Serious arguments with their partner were cited by 14.5% of the female students and 15.1% of the male students, a difference that was not statistically significant: χ^2^(1, *N* = 800) = 0.06, *p* = 0.800. Personal illness was the least frequently cited problem and was mentioned by 12.6% of the female students and 9.5% of the male students, a difference that was not statistically significant: χ^2^(1, *N* = 800) = 0.19, *p* = 0.170. When analysing whether there were differences in the frequency with which such stressors occurred between the two time periods studied, statistically significant differences were only found in two of them: illness of relatives and/or loved ones, which was cited by 33.5% of male students and female students in the new normality period and 24.8% in the lockdown period (χ^2^(1, *N* = 800) = 7.42, *p* = 0.006); and death of relatives and/or loved ones, which was cited by 23.3% of the sample in the new normality period and by 15.5% in the lockdown (χ^2^(1, *N* = 800) = 7.69, *p* = 0.006).

Two years later, no stressful event presented significant differences between female and male students. The most frequently stressful event cited was illness of relatives and/or loved ones, which was reported by 52.6% of female students and 50% of male students; the second was economic problems, being cited by 42.7% of female students and 39.2% of male students. Coming in third and four place were serious arguments with one’s family and the death of one or more relatives or loved ones, which presented similar percentages: 34.6% for female students and 36% for male students and 36% for female students and 33.3% for male students, respectively. The remaining two stressful events cited were personal illness, as reported by 23.7% of female students and 27.5% of male students, and serious arguments with their partner, which was cited equally by female and male students at 19%.

### 3.2. Risk and Protective Factors That Predict Psychological Distress

The main results of the multiple regression predicting psychological distress in male students and female students in the lockdown period are shown in Table 1; those for the new normality period corresponding to October 2020 are shown in Table 2; and the results for the two years after the first wave of the pandemic period are shown in Table 3. As can be seen, Model 1, including age as a predictor, was not statistically significant at any time except for the two-years-later time period in female students, remaining significant with the inclusion of social support and stressful events but losing significance with the inclusion of self-esteem. When social support was included in Model 2, the change in *R*^2^ identified that social support significantly affected the psychological distress of male students and female students in the three time periods studied, presenting small effect sizes during the lockdown and the new normality period and medium ones during the two-years-later period. The variable stressful events associated with COVID-19 were introduced in Model 3, producing a statistically significant increase in the prediction of psychological distress with medium effect sizes, but the increase in the explained variance was just 3% in the two-years-later period for both male and female students. It seems that at this time, stressful events related to COVID-19 had a damped effect. The statistical power in Model 3 greatly exceeded 80% during the lockdown and new normality period. In the case of the two-years-later period, this threshold was surpassed in Model 1 for female students and in Model 2 for male students. The inclusion of self-esteem in Model 4 also resulted in statistically significant (*p* < 0.001) increases in the prediction of psychological distress. The beta values in this final model, with all the variables in the equation, showed that for all periods and genders, the most relevant variable was self-esteem, where higher self-esteem predicted lower levels of psychological distress. The statistical power was 1, and the effect sizes were large for male and female students. The second most relevant variable in the prediction of psychological distress was the number of stressful events, with greater distress seen in those who had experienced a higher number of stressful events only during the lockdown and new normality period. In the case of the two-years-later period, the relevance of stressful events and social support was lower than in the previous time periods measured. The percentage of variance explained in male students in the lockdown period was 27% and in female students 36%, whilst in the new normality period, it was 33% in male students and 36% in female students, and finally, two years later, it was 46% in male students and 39 in female students.

## 4. Discussion

The objective of this study was to find out how the COVID-19 pandemic impacted university students in Spain in three different periods and to analyse the gender differences that could have occurred in psychological distress and stressful events. Additionally, the protective role of social support and self-esteem was analysed.

The results of our study show that the prevalence of psychological distress was higher in female than in male students in absolute terms (55.9% vs. 44.1%), confirming studies carried out in other countries, where it was also found that women showed higher psychological distress and symptomatology than men during the pandemic [16,34]. The results obtained are remarkable in that the female university students in our sample, in the first wave of COVID-19, presented a psychological distress prevalence similar to that of healthcare workers or medical staff, who at this time of the pandemic did not have adequate protection means and were in close contact with infected patients. Rodríguez-Jiménez et al. [52] found that 74.9% of healthcare workers suffered clinical levels of psychological distress during the first wave of the pandemic in Spain, using the same measure as that used in our study, GHQ-12, but with a cut-off score of 3, which is lower than the more restrictive cut-off score of 4 used in our work, which allowed a greater number of healthcare workers to be included. High psychological distress was seen at a prevalence that is similar to what we found in female students, reaching 74.2% when more restrictive criteria were used. The psychological distress levels in female students found in the present work was also higher than that found by two other studies that used the same questionnaire and scoring method, that by Rens et al. [53], who found that 56.82% of men and 67.92% of women had psychological distress in a study carried out in Belgium with a young population, and one by Pierce et al. [9], in a study performed in the United Kingdom in April 2020, during the first wave of COVID-19, who found a prevalence of 44% in 16–24-year-old women. On the other hand, the prevalence of psychological distress in male students in our study, 57.7%, was similar to that reported in Rens et al. [53]. However, the hypothesis that women would exhibit higher levels of psychological distress than men was only confirmed for the two time periods corresponding to the first wave of the pandemic itself, the “lockdown” and “new normality” periods, and not in the period corresponding to two years after the pandemic, when male and female students showed similar levels of psychological distress.

The level of distress was very similar between the two time periods of the first wave of COVID-19: the lockdown, where 73.9% of female students and 55.6% of male students had psychological distress, and months later, during the new normality period, where the percentages were 74.4% of female students and 59.8% of male students. During the period of time corresponding to two years later, an important decrease was observed, with percentages of 51.2% for female students and 52.4% for male students. Here, it should be highlighted that the decrease found for male students (3.2–7.4%) was modest compared with the important decreases presented by female students (21.7–23.2%). These results represent a clear recovery to pre-pandemic levels of psychological distress, especially by female students. The study with the closest similarity to the one performed in this work is that of Arias-de la Torre et al. [54], conducted just before the pandemic, which assessed psychological distress in university students from nine Spanish universities using the same questionnaire but a more permissive cut-off period of 3, obtaining a prevalence of 46.9% for male students and 54.2% for female students, percentages close to those obtained in this study, two years after the first wave of COVID-19.

With respect to the second hypothesis in our study, where a consecutive downward trend was expected from the lockdown to the new normality period to the two-years-later period, given that the percentages did not significantly change in the two time periods analysed during the first wave of COVID-19, we cannot confirm this hypothesis. The two-years-later time period showed the lowest psychological scores, as was expected, but the predicted slope from the lockdown to the two-years-later period did not happen; instead, we observed an increase in psychological distress levels at the intermediate time period of the new normality stage. The reason why students did not show a lower level of psychological distress during the new normality period, when educational centres were reopened, face-to-face teaching was resumed, and restrictions were minimal compared with the strict restrictions during the lockdown period, is not known, but it could be related to the greater number of stressful events reported in the new normality period, mainly the illness and death of relatives and loved ones, and this takes us to the third hypothesis.

The students who had experienced more stressful events related to the pandemic in the first wave of COVID-19 reported more psychological distress, a result that indicates that directly suffering negative events of the pandemic leads to a greater risk of psychological distress. Studies carried out with students in other countries have also found an association between a greater number of stressful events during the COVID-19 pandemic and more serious mental symptoms [22]. As mentioned before, the students in our sample reported a higher number of stressful events, specifically a higher frequency of illness and/or death of loved ones in the “new normality” period in October 2020 than in the “lockdown” period, and that could explain why the levels of psychological distress in our sample did not drop in the less restrictive “new normality” period. Also, female students reported having suffered a greater number of stressful events during the COVID-19 pandemic than male students, in agreement with results of other studies, where a higher risk of stress was found in women than in men [8,55], which could help to explain the higher psychological distress found in female students in comparison to male students during these time periods. The significant decrease in psychological distress two years later completely confirms our third hypothesis. As shown in Figure 1, female students showed this effect, with the same percentages of having experienced more than one stressful event related to the pandemic and presenting scores equal or higher than 4 in psychological stress. The most frequent stressful events in each period of time reflected the specific situation in regard to the pandemic wave or phase; in the first time periods, lockdown and new normality, serious arguments with one’s family was the most cited stressful event, reflecting the lack of movement and the isolation of the family at home, with no possibility of even taking a walk outside in the lockdown, which could have led to greater friction and discussions at the family level. The new normality period, which could have been a respite, based on data on stressful events and psychological distress in this study, appears to have gotten worse rather than better, with higher percentages of stressful and psychological distress. The most common stressful event reported two years after the first wave of the COVID-19 pandemic was the illness of relatives and/or loved ones, reflecting the high proportion of people who were infected with COVID-19 in Spain, with more than thirteen million cases officially declared from 3 January 2020 to 6 September 2023 [56].

The fourth hypothesis proposed that experiencing a higher number of stressful events related to COVID-19 would predict higher psychological distress. Our results confirm this hypothesis for the first wave of the pandemic, in both time periods, lockdown and new normality, but its predictive power is lower two years later, mainly when self-esteem is included in the analysis. The high ability of stressful events to predict psychological distress confirms studies conducted during the pandemic, where negative experiences associated with COVID-19 were shown to be the best predictors of psychological distress, especially in women and young people [22,57].

The roles of high social support and self-esteem in predicting lower psychological distress represent the fifth and sixth hypotheses, respectively. Our results have confirmed that self-esteem had an important protective role against psychological distress in each time period, as reported in previous studies [58], fully confirming the sixth hypothesis; but the protective role of social support seems to be more limited. Our results only partially agree with those of other studies, in which social support was shown to be associated with lower psychological distress during the COVID-19 pandemic [2,34,59]. However, social support as a variable with an important role in predicting psychological distress was surpassed when stressful events and self-esteem enter the equation in the first wave of COVID-19. The higher relevance of self-esteem over social support could be explained by the fact that the best predictor of self-esteem is social support in the developmental stage of emerging adulthood [36,37]. Therefore, although social support is important, its predicting role is shadowed by the role of self-esteem.

Summarising, the effect of stressful events associated with the first wave of COVID-19 in Spanish university students resulted in a higher impact than expected, mainly in female students, with levels of psychological distress similar to those reported for health workers. These high levels of psychological distress seem to be directly associated with stressful events related to the pandemic and the measures taken to control it, where the most frequently reported events were serious arguments with one’s family, illness of relatives and/or loved ones, and economic problems. Although we expected lower levels of psychological distress in the new normality period in comparison with the strict lockdown period, due to the relaxation of restrictions and the return to classrooms, our results show similar psychological distress levels in both periods. This is probably related to the higher number of stressful life events, including illnesses and deaths of relatives and/or loved ones, reported by the sample that answered the questionnaires in the new normality period compared with the sample that answered the questionnaires in the lockdown period. Here, it should be highlighted that being an emerging adult, as the students of our samples were, and being a woman were predicative of a worse outcome in psychological distress during the first phases of the pandemic, confirming studies performed in different countries [16,22,23,34,47], including in Spain [13,14], where the highest psychological impact of the COVID-19 pandemic was observed for young people and women. On the other hand, in the two-years-later period, we found that psychological distress had returned to levels similar to pre-pandemic levels. Although the number of stressful events increased throughout the pandemic, with a median for both males and females of 2, as compared to the median of 1 in the first wave of COVID-19, the passage of time seems to have allowed students to adapt to stressful situation of the pandemic and return to pre-pandemic psychological distress levels. Finally, it is worth noting the role of social support and, with a stronger effect, self-esteem in the reduction in psychological distress.

Finally, our study had four main limitations. The main one is that it was a repeated cross-sectional study, with questionnaires answered during three different time periods in three independent samples, so causal inferences cannot be made. Also, this study did not use a random sample, so it may not be representative of all Spanish students, and finally, the information was gathered using self-reports, a method that may have biases such as social desirability. Finally, a measurement limitation should be considered in the case of the stressful events related to COVID-19, with economic problems, arguments with relatives, illnesses, and deaths being included without any ponderation by the participants. Further studies should use random samples, carry out a multi-method evaluation, and be longitudinal. The inclusion of more time periods during the pandemic could enhance the knowledge of risk and protective factors of psychological distress. Despite these limitations, our work highlights the high level of psychological distress and, therefore, the risk of mental health problems that students suffered during the COVD-19 pandemic, especially in the case of women.

## 5. Conclusions

The present work shows that the first wave of the COVID-19 pandemic represented a significant risk for the mental health of students, a risk that was greater in females than in males and that did not seem to be merely temporary and associated with the closure of schools and universities. Experiencing a greater number of stressful life events was a risk factor for psychological distress, whilst high self-esteem and, secondarily, high social support, have been shown to be protective factors.

Given the important role of self-esteem and social support as protective factors against stress, both variables should have a central role in any intervention and be promoted from early childhood. As for social support, apart from the development of social networks in universities, it is important to ensure the availability of psychological services that students could turn to. Health and care professionals must be aware of the higher susceptibility of women to stressful life events and their resultant higher levels of stress in comparison with men. Women are at a higher risk when faced with stressful situations, and therefore in need of more dedicated and extended support, something that must be considered when allocating resources.

Our study showed the importance that self-esteem and social support had as protective factors against psychological distress during the pandemic. Self-esteem should be fostered from childhood so that students are better equipped to deal with stressful situations.

## Figures and Tables

**Table 1:** Hierarchical regression results for psychological distress in male and female students in the lockdown period in Spain (*n* = 400).

	Model 1			Model 2			Model 3			Model 4		
Variable	*B*	*SE B*	β	*B*	*SE B*	β	*B*	*SE B*	β	*B*	*SE B*	β
MALE (*n* = 189)												
Age	0.16	0.19	0.06	0.21	0.18	0.08	0.27	0.18	0.11	0.43	0.17	0.12
Social Support				−0.28	0.06	−0.32 ***	−0.25	0.06	−0.29 ***	−0.11	0.06	−0.14 *
Stressful Events							1.02	0.38	0.19 **	0.77	0.35	0.14 *
Self-Esteem										−0.53	0.09	−0.41 ***
*R* ^2^		0.01			0.11			0.14			0.27	
*F* for change in *R*^2^		0.74			21.33 ***			7.23 **			33.34 ***	
1 − β		0.55			0.87			1			1	
*f* ^2^		0.04			0.12			0.16			0.37	
FEMALE (*n* = 211)												
Age	−0.21	0.20	−0.07	−0.20	0.19	−0.07	−0.27	0.18	−0.09	−0.00	0.17	0.00
Social Support				−0.22	0.06	−0.25 ***	−0.13	0.06	−0.15 *	−0.02	0.05	−0.03
Stressful Events							2.20	0.36	0.39 ***	1.62	0.34	0.29 ***
Self-Esteem										−0.60	0.09	−0.44 ***
*R* ^2^		0.01			0.07			0.21			0.36	
*F* for change in *R*^2^		1.09			13.41 ***			37.31 ***			48.48 ***	
1 − β		0.66			0.63			1			1	
*f* ^2^		0.01			0.07			0.27			0.56	

* *p* < 0.05; ** *p* < 0.01; *** *p* < 0.001; 1 − β = statistical power; *f*^2^ = effect size.

**Table 2:** Hierarchical regression results for psychological distress in male and female students in the new normality period in Spain (*n* = 400).

	Model 1			Model 2			Model 3			Model 4		
Variable	*B*	*SE B*	β	*B*	*SE B*	β	*B*	*SE B*	β	*B*	*SE B*	β
MALE (*n* = 189)												
Age	−0.04	0.22	−0.01	−0.06	0.22	−0.02	−0.05	0.21	−0.02	0.06	0.19	0.02
Social Support				−0.16	0.07	−0.18 *	−0.13	0.06	−0.14 *	0.05	0.06	0.06
Stressful Events							1.62	0.39	0.29 ***	1.44	0.34	0.26 ***
Self-Esteem										−0.73	0.10	−0.51 ***
*R* ^2^		0.00			0.03			0.12			0.33	
*F* for change in *R*^2^		0.03			6.41 *			17.39 ***			58.48 ***	
1 − β		0.00			0.35			0.88			1	
*f* ^2^		0.00			0.03			0.14			0.49	
FEMALE (*n* = 211)												
Age	0.18	0.24	0.05	0.06	0.23	0.02	0.11	0.22	0.03	0.17	0.19	0.05
Social Support				−0.24	0.07	−0.24 ***	−0.20	0.07	−0.20 **	−0.03	0.06	−0.03
Stressful Events							1.45	0.31	0.30 ***	1.30	0.27	0.27 ***
Self-Esteem										−0.69	0.08	−0.50 ***
*R* ^2^		0.03			0.06			0.15			0.36	
*F* for change in *R*^2^		0.55			12.13 ***			21.51 ***			70.03 ***	
1 − β		0.97			0.59			0.98			1	
*f* ^2^		0.03			0.06			0.18			0.56	

* *p* < 0.05; ** *p* < 0.01; *** *p* < 0.001; 1 − β = statistical power; *f*^2^ = effect size.

**Table 3:** Hierarchical regression results for psychological distress in male and female students two years after COVID-19 pandemic period in Spain (*n* = 400).

	Model 1			Model 2			Model 3			Model 4		
Variable	*B*	*SE B*	β	*B*	*SE B*	β	*B*	*SE B*	β	*B*	*SE B*	β
MALE (*n* = 189)												
Age	−0.29	0.22	−0.01	−0.37	0.20	−0.12	−0.42	0.20	−0.14 *	−0.22	0.17	−0.07
Social Support				−0.31	0.05	−0.40 ***	−0.29	0.05	−0.38 ***	−0.10	0.04	−0.13 *
Stressful Events							0.55	0.27	0.14 *	0.23	0.23	0.06
Self-Esteem										−0.73	0.08	−0.59 ***
*R* ^2^		0.01			0.17			0.19			0.46	
*F* for change in *R*^2^		1.75			35.84 ***			4.09 *			91.40 ***	
1 − β		0.28			1			1			1	
*f^2^*		0.01			0.21			0.24			0.85	
FEMALE (*n* = 211)												
Age	0.79	0.25	0.22 **	0.70	0.23	19 **	0.61	0.23	0.17 **	0.30	0.20	0.08
Social Support				−0.32	0.06	−0.32 ***	−0.25	0.07	−0.25 ***	−0.06	0.06	−0.06
Stressful Events							0.92	0.30	0.21 **	0.52	0.26	0.12 *
Self-Esteem										−0.68	0.08	−0.52 ***
*R* ^2^		0.05			0.15			0.18			0.39	
*F* for change in *R*^2^		10.25 **			24.38 ***			9.55 **			69.70 ***	
1 − β		0.91			1			1			1	
*f* ^2^		0.05			0.18			0.22			0.64	

* *p* < 0.05; ** *p* < 0.01; *** *p* < 0.001; 1 − β = statistical power; *f*^2^ = effect size.

## Data Availability

Data presented in this study are not readily available due to ethical restrictions. Reasonable requests to access the data can be made to the corresponding author.
